# Handheld optical coherence tomography for clinical assessment of dental plaque and gingiva

**DOI:** 10.1117/1.JBO.25.11.116011

**Published:** 2020-11-26

**Authors:** Jungeun Won, Pin-Chieh Huang, Darold R. Spillman, Eric J. Chaney, Ralf Adam, Malgorzata Klukowska, Ronit Barkalifa, Stephen A. Boppart

**Affiliations:** a University of Illinois Urbana–Champaign, Beckman Institute for Advanced Science and Technology, Urbana, Illinois, United States; b University of Illinois Urbana–Champaign, Department of Bioengineering, Urbana, Illinois, United States; c Procter & Gamble Company, Clinical Operations, German Innovation Center, Kronberg, Germany; d Procter & Gamble Company, Oral Health Science Center, Mason, Ohio, United States; e University of Illinois Urbana–Champaign, Department of Electrical and Computer Engineering, Urbana, Illinois, United States; f University of Illinois Urbana–Champaign, Carle Illinois College of Medicine Champaign, Illinois, United States

**Keywords:** biofilm, dental plaque, gingiva, optical coherence tomography, oral cavity

## Abstract

**Significance:** Optical coherence tomography (OCT) offers high spatial resolution and contrast for imaging intraoral structures, yet few studies have investigated its clinical feasibility for dental plaque and gingiva imaging *in vivo*. Furthermore, the accessibility is often limited to anterior teeth due to bulky imaging systems and probes.

**Aim:** A custom-designed, handheld probe-based, spectral-domain OCT system with an interchangeable attachment was developed to assess dental plaque and gingival health in a clinical setting.

**Approach:** Healthy volunteers and subjects with gingivitis and sufficient plaque were recruited. The handheld OCT system was operated by trained dental hygienists to acquire images of dental plaque and gingiva at various locations and after one-week use of oral hygiene products.

**Results:** The handheld OCT can access premolars, first molars, and lingual sides of teeth to visualize the plaque distribution. OCT intensity-based texture analysis revealed lower intensity from selected sites in subjects with gingivitis. The distribution of the dental plaque after one-week use of the oral hygiene products was compared, showing the capability of OCT as a longitudinal tracking tool.

**Conclusions:** OCT has a strong potential to display and assess dental plaque and gingiva in a clinical setting. Meanwhile, technological challenges remain to perform systematic longitudinal tracking and comparative analyses.

## 1 Introduction

Dental plaque, a bacterial biofilm constantly forming on surfaces within the mouth, is responsible for dental caries and periodontal diseases.^1^ When soft dental plaque is not removed, the dental plaque may develop into calculus (hardened plaque) under or above the gumline. The presence of plaque and calculus can irritate the gingiva (or simply, the gums), leading to the gingival inflammation called gingivitis.^2^^,^^3^ The common symptoms of gingivitis include swollen, bleeding, and receding gingiva. Gingivitis is one of the most common oral diseases worldwide, and around 56% of adults in the United States have mild to severe gingivitis.^4^ If gingivitis is left untreated, gingivitis can advance to periodontitis, tooth loss, and other severe oral diseases.

The current diagnosis and evaluation of dental plaque and gingivitis rely on the visual examination and the patient’s dental and medical history.^3^ The dental plaque can be qualitatively assessed using plaque-disclosing products, such as disclosing solution or tablets based on iodine, gentian violet, or erythrosine, which stain plaque a bright color.^5^ Surface dental plaque can also be revealed with fluorescence imaging as well.^6^^,^^7^ However, these methods are often confined to tissue surfaces, and thus may not provide detailed information when monitoring the therapeutic effects and the disease progression. One method to examine severe gingival conditions is to measure the pocket depth using a periodontal probe inserted into the gum pocket. Although periodontal probing provides information about the attachment of the gingiva to the tooth, it is uncomfortable and painful.^8^ Several noninvasive imaging modalities, such as dental x-rays, x-ray computed tomography, magnetic resonance imaging, and ultrasound imaging, have been actively investigated as a potential, improved monitoring method,^9^ emphasizing the need for advanced higher-resolution imaging techniques to detect subtle quantitative changes.

Optical coherence tomography (OCT)^10^ collects backscattered light from the tissue and generates depth-resolved, cross-sectional images with an axial resolution of 2 to 10  μm. Dental OCT has shown promising results with improved spatial resolutions over standard imaging modalities, and several review articles are also available.^11^^–^^14^ Both hard tissues and soft tissues have been investigated to study tooth decay,^15^^,^^16^ enamel cracks,^17^ restoration evaluation,^18^ calculus,^19^ pulp chamber,^20^ oral cancers,^21^^,^^22^ and periodontal diseases.^23^^,^^24^ Although OCT can assess various features in the oral cavity, challenges exist for the further clinical translation of OCT technologies. Most commercial OCT systems are bulky and not designed for oral cavity imaging, making accessibility limited to largely frontal views of anterior teeth (incisors and canine). Only a few studies have showed the capability of imaging up to the premolars.^25^^,^^26^ Several research groups have developed customized systems for dental imaging, ranging from handheld probes^27^^–^^29^ to endoscope-based designs.^30^ However, many studies are yet limited to *ex vivo* tissues and *in vitro* samples,^16^^,^^31^ or *in vivo* imaging from volunteers in laboratory settings.^28^^,^^30^ In addition, the capability of OCT as a longitudinal tracking tool has not been investigated for assessing dental plaque and gingivitis, and only a few studies have examined the capability in tooth composites.^25^^,^^26^^,^^32^

In this paper, we present a custom-designed, handheld probe-based, portable spectral-domain OCT system developed and demonstrated for dental plaque and gingiva imaging in a clinical setting. The handheld OCT probe was operated by trained dental hygienists on human subjects with mild to moderate gingivitis, with sufficient dental plaque. Feasibility of OCT imaging of both the anterior and posterior teeth, as well as the buccal and lingual sides, was explored. Furthermore, longitudinal tracking of dental plaque was performed to observe the effects of brushing products. Lastly, perspectives on designing clinical oral OCT systems are thoroughly discussed for future system development and studies.

## 2 Materials and Methods

### 2.1 Handheld OCT System for Oral Imaging

**Fig. 1:**
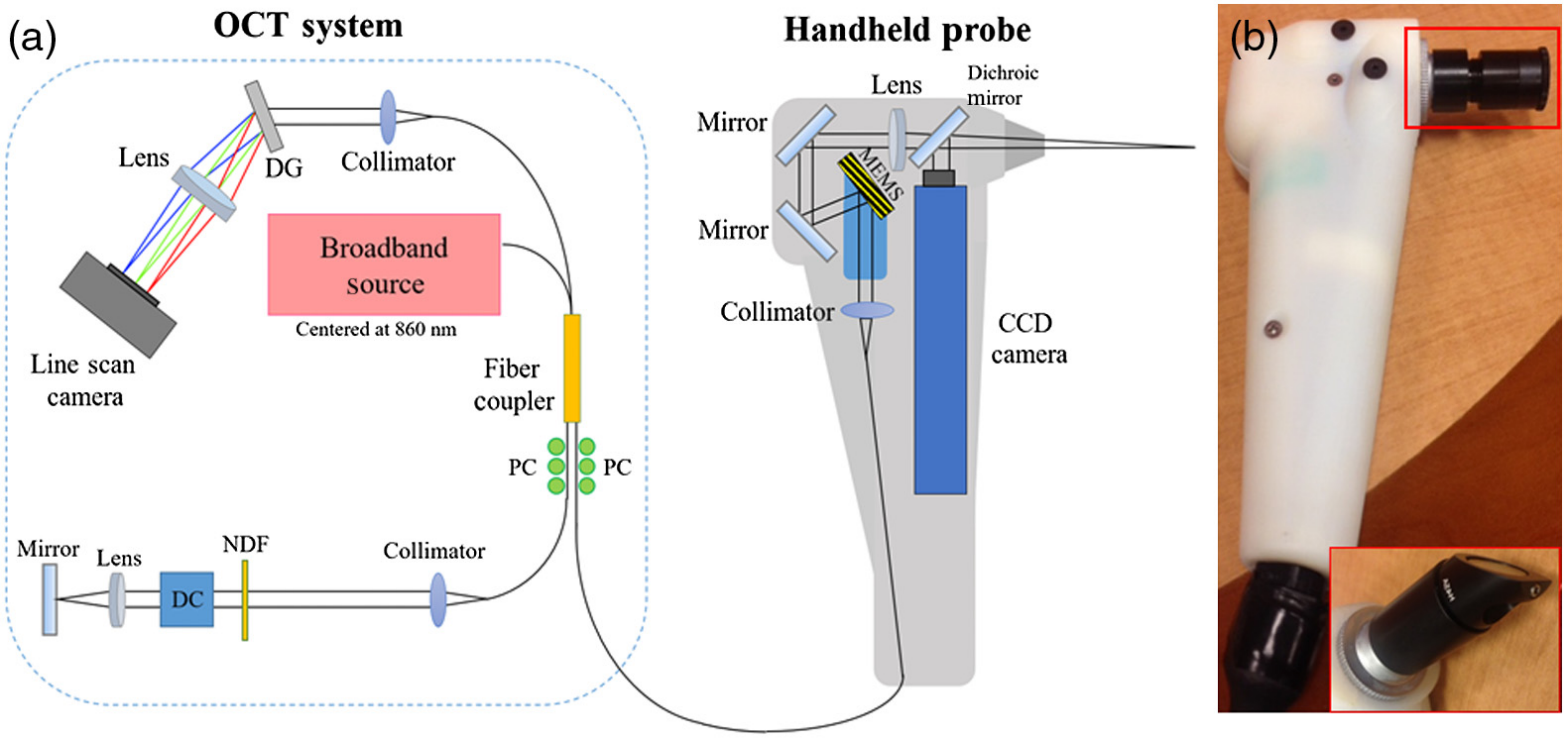
The handheld OCT probe for imaging the human oral cavity. (a) Schematic diagram of the handheld SD-OCT system and (b) photo of the probe. The attachments (top: straight and bottom: 45-deg mount) in the red box are interchangeable depending on the regions of interest. CCD, charge-coupled device; DC, dispersion compensator; DG, diffraction grating; NDF, neutral density filter; PC, polarization controller.

The handheld OCT system used in this study for oral imaging was adapted from previous designs for primary care and middle ear imaging^33^^,^^34^ shown in Fig. 1. The custom-developed spectral-domain OCT system employed a superluminescent diode (SLD-351-HP2, Superlum, Ireland) centered at 860 nm with a spectral bandwidth of 130 nm. The axial and transverse spatial resolutions of the system were ∼7 and 25  μm in air. The system operated at a 32-kHz A-scan rate (Cobra 800, Wasatch Photonics, NC) with a B-scan rate of ∼20  Hz and an imaging depth of 2.8 mm. A MEMS scanner (AdvancedMEMS, CA) was used to provide a lateral scanning range and a field-of-view (FOV) of 3.3 mm. The simultaneous visualization of the sample was enabled by a CCD camera (MU9PC-MH, XIMEA, CO) in order to guide the OCT beam during imaging. The power incident on the sample was measured around 2.6 mW. The signal-to-noise ratio (SNR) was ∼100  dB with a perfect reflector in the sample arm. The objective lens had a focal length of 50 mm. The probe could work either in contact or non-contact mode by adjusting the lens tube (SM05, Thorlabs, NJ, see red box in Fig. 1). In addition, a mirror in a 45-deg mount (H45, Thorlabs, NJ) was used to visualize the lingual sides of teeth and the buccal sides of posterior teeth. The dimension of the probe without the adjustable lens tube was around 6  in.×2  in.×1  in. (15.2  cm×5.1  cm×2.5  cm, height×width×depth). The adjustable lens tube had a diameter of a half inch (1.3 cm).

### 2.2 Oral OCT Imaging in Clinical Setting

A study protocol was approved by both Institutional Review Boards (IRBs) at the University of Illinois Urbana-Champaign and the Chesapeake IRB for the Procter & Gamble Company (P&G). The OCT system was built at the Biophotonics Imaging Lab in Urbana-Champaign, Illinois and transported to the Oral Health Science Center (OHSC) in Mason, Ohio, for the clinical study. Informed consents were obtained from all participants prior to imaging. Images at various locations in the oral cavity were acquired from healthy adult volunteers. Then a total of 10 subjects with mild to moderate gingivitis and with sufficient dental plaque were recruited at OHSC after the standard oral examination performed by a licensed examiner. The control measurements were taken from the healthy gingiva regions of the subject group, determined by the licensed examiner. Around two to three targeted imaging sites per subject were assigned.

Subjects refrained from oral hygiene the morning of the visit for OCT imaging (week 1: first visit for OCT imaging) and also refrained from eating, drinking, chewing gum, and tobacco for 4 h prior to the appointment time. During the visit, OCT imaging was performed by trained dental hygienists in a clinical exam room. Prior to imaging, the hygienists were trained for ∼1  h on manipulating the probe and understanding OCT images. Training was performed by the OCT expert who was involved in developing the OCT system. Around 20% of the total number of subjects, corresponding to 2 subjects, were imaged during the training/calibration session. For each site, an average of 15 to 30 min was allowed for OCT imaging. Approximately 200 OCT images were collected from each site. The subjects were asked to lie down on a dental chair, as in standard dental procedures. Standard room lighting, typical for a dental exam room, was used. The dental light above the head/mouth was tilted and adjusted for each patient case so that the light was incident directly into the oral cavity to provide adequate illumination for the CCD images. The computer monitor with the OCT device was also turned on, with standard brightness and contrast. No bite block or marking was utilized during the study. For efficient and rapid sanitization of the probe between subjects, a sterile, disposable plastic sheath originally designed for ultrasound imaging (Spectrum Laboratories Inc., CA) was used to wrap around the entire probe using a sterilized rubber band, as in standard procedures for probe-based ultrasound and OCT imaging in intraoperative settings.^35^

Next, subjects were randomly assigned to one of the two groups (five subjects per group): product and control group. The product group was instructed to use products (toothpaste, power toothbrush, mouth rinse, and floss) twice a day for 7 days after appropriate training to use the products, whereas the control group was instructed not to perform any oral hygiene for 7 days. On the next visit (after 7 days, week 2), previously assigned sites for all subjects were imaged with OCT (same imaging procedure as in week 1) to monitor the changes in dental plaque and gingival conditions.

### 2.3 Analytical Method

Previous studies have demonstrated that the inflamed gingiva tends to have a lower intensity in OCT.^36^ To quantitatively examine the severity of the gingival inflammation, texture analysis^37^ was performed based on the speckle intensity to assess the spatial-dependent homogeneity of the tissue. A region of interest (ROI) at around 100 to 200  μm in depth was selected with the size of 100×150  pixels, corresponding to around 290  μm×520  μm (depth×width). The OCT intensity (before log-compression) within the ROI was fitted to the gamma distribution, and the shape parameter (α) and the scale parameter (β) of the fitted gamma distribution were determined as follows:

$$f(x,\alpha ,\beta )=\frac{1}{\Gamma (\alpha )}\beta^{\alpha }x^{\alpha -1}e^{-\beta x},$$ (1)

where x is the intensity distribution within the ROI. The ratio of the shape parameter to the scale parameter (α/β) was computed for each ROI, which may relate to the effective tissue scatterer density.^37^^,^^38^ As the ROI was spatially moved across the gingival region in each frame, the mean ratio (α/β) was determined. The statistical comparison was performed using Welch’s t-test with a significance level of 0.05.

## 3 Results

### 3.1 Handheld OCT for Soft and Hard Tissue Imaging in the Human Oral Cavity

**Fig. 2:**
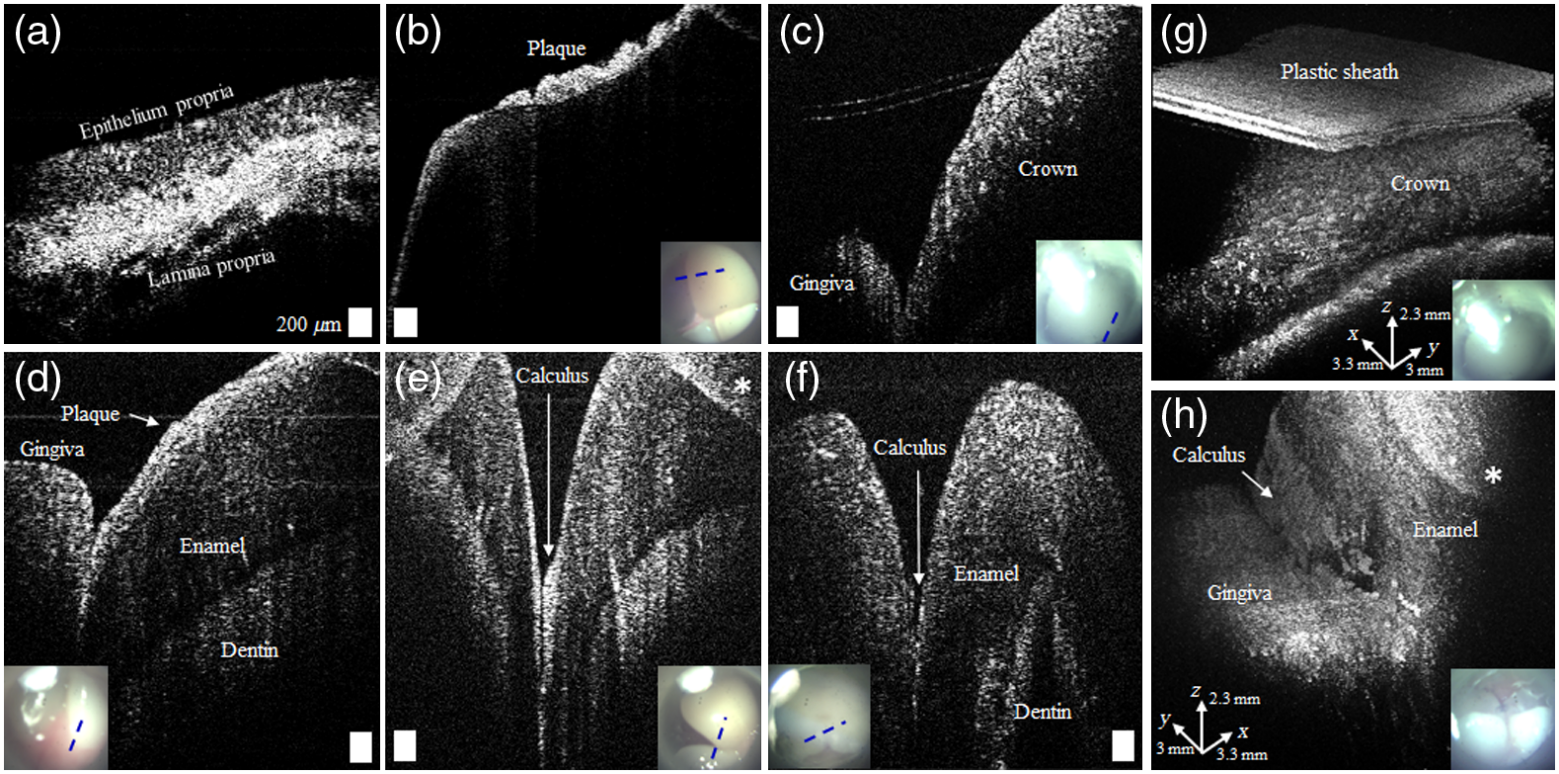
OCT oral cavity imaging on healthy volunteers. Representative images of (a) oral mucosa; (b) dental plaque on an incisor; (c) dental crown on a premolar; (d) junction of gingiva and incisor; (e) calculus between canine and premolar teeth; (f) lingual view of an incisor; (g) 3D visualization of the dental crown in (c); and (h) 3D visualization of the lingual side of a molar with calculus. The inset images represent surface views of teeth, with the blue dashed line indicating the scanned region. Scale bars represent 200  μm. Asterisks indicate a region with mirror artifacts.

Prior to subject recruitment for the longitudinal study, oral cavity imaging using the handheld OCT probe was performed on healthy volunteers. As shown in Fig. 2, the handheld OCT can visualize hard tissue (enamel and dentin) as well as soft tissue (oral mucosa, gingiva, and dental plaque). It is also noted that the lingual sides [Figs. 2(f) and 2(h)], premolars [Figs. 2(c) and 2(e)], and molars [Fig. 2(h)] are accessible with the 45-deg attachment. Dental plaque was readily visible on the top surface of the enamel [Fig. 2(b)], and the presence of the hardened plaque (calculus) was often detected at the junction between gingiva and enamel [Fig. 2(d)] and between teeth [Fig. 2(e)]. Notably different scattering from the dental crown was also observed in Fig. 2(c). The inset photos illustrate the simultaneous surface visualization of the imaged regions. A three-dimensional view from both the buccal and lingual sides [Figs. 2(g) and 2(h)] is also provided by the handheld OCT system. Note that mirror image artifacts caused from the Fourier transformation used to process OCT images were visualized in some images due to the limited imaging depth. As a result of these mirror artifacts, the image may appear to be folded at the top of the image. To avoid this artifact, the user needs to properly position the desired tissue region within the imaging range of the OCT system.

### 3.2 OCT Visualizes the Presence of Dental Plaque at Various Sites

**Fig. 3:**
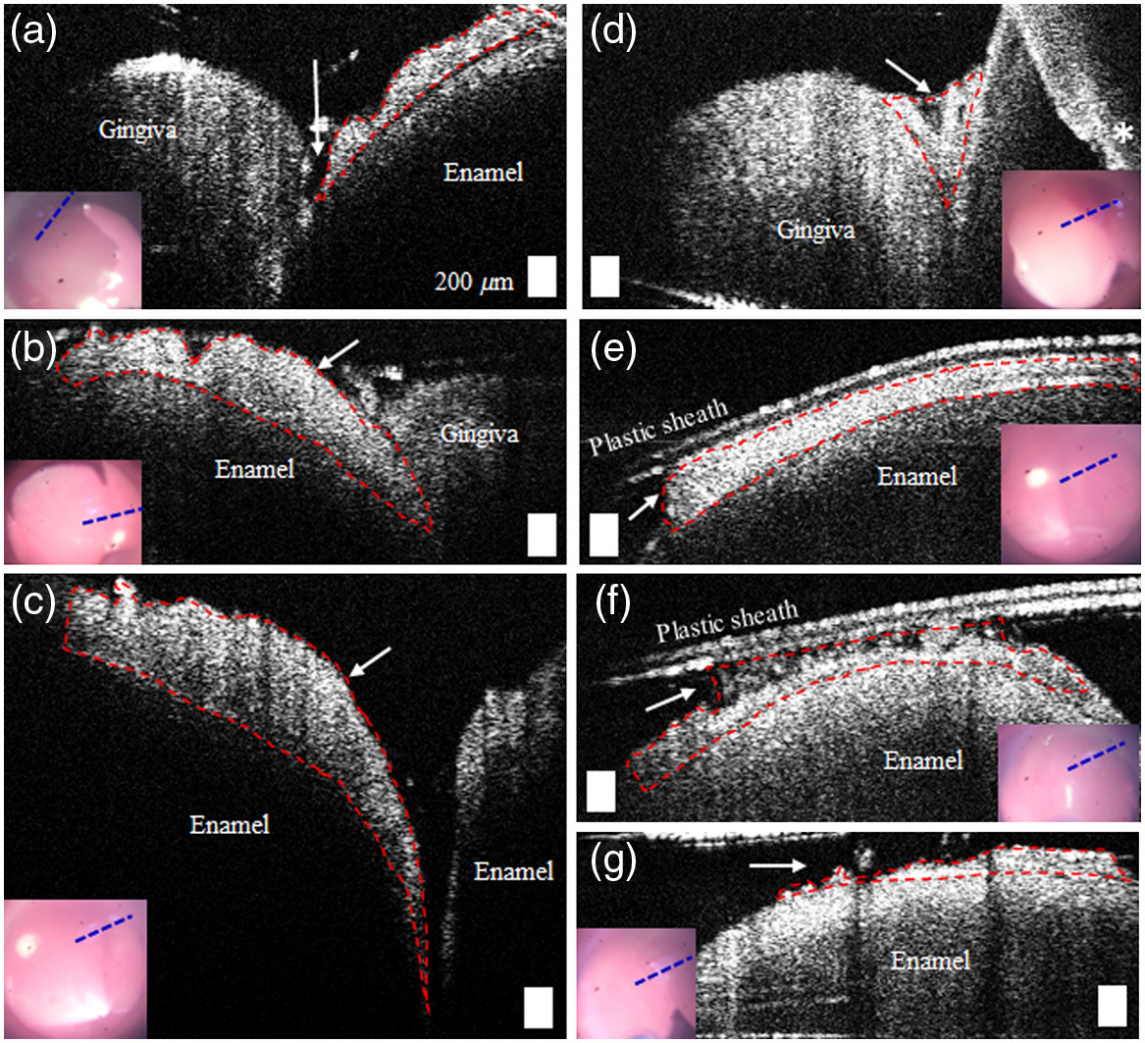
OCT images of dental plaque at various locations. The red dashed lines show an approximate region with plaque. The inset video images indicate the simultaneous surface view of the sites, and the blue dashed lines show the scanned regions. (a)–(c) The presence of the dental plaque on the surface of the enamel and (d) the junction between gingiva and enamel. (e)–(g) The disrupted dental plaque due to the plastic sheath during imaging. (a), (d), and (e) were obtained from frontal incisors, whereas (b), (c), (f), and (g) were acquired from fontal canines. Scale bars represent 200  μm. Asterisk indicates a region with mirror artifacts.

The representative images of the dental plaque from the recruited subjects are shown in Fig. 3. The dental plaque on the enamel, with red dashed lines indicating a boundary, is shown in Figs. 3(a)–3(c). The red lines were manually determined based on the intensity. Figure 3(d) visualizes the small amount of the dental plaque in between gingiva and enamel. Figures 3(e)–3(g) demonstrate a case where the plaque was disrupted during imaging, as the plastic sheath contacted the plaque. This also confirms that the dental plaque on the surface of the enamel is likely the soft dental plaque. A wide range in the thickness of the plaque was observed, from around 50 to 350  μm.

### 3.3 Free Gingiva Imaging Comparing Healthy and Gingivitis Conditions

**Fig. 4:**
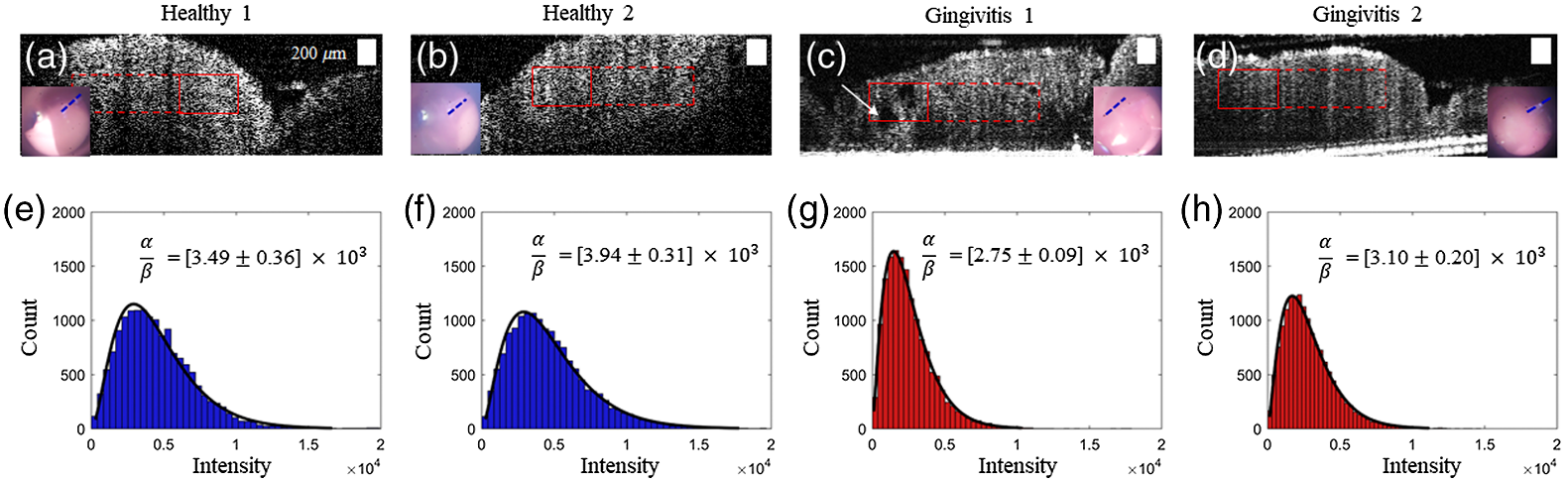
OCT images of gingiva from regions of (a), (b) healthy sites and (c), (d) gingivitis sites. The red solid box represents the ROI, and the corresponding fitting results to the gamma distribution are shown in (e)–(h). The red dashed box indicates the spatial sliding of the box to compute the mean ratio, α/β. (a)–(d) were obtained from the buccal sides of a premolar. The line of fit in (e)–(h) is in solid black. The inset images are the simultaneously acquired surface views, with the blue dashed lines indicating the scanned regions. Scale bars represent 200  μm.

The representative gingiva images from the healthy sites and sites with gingivitis are shown in Fig. 4. There were no consistent differences between healthy and gingivitis sites, except a few cases where OCT captured ulcerative lesions (white arrows) in gingiva, as shown in Fig. 4(c). In order to quantitatively compare the gingival inflammation, texture analysis was performed on the selected ROIs (red boxes). Representative results from fitting the gamma distribution are shown in Figs. 4(e) to 4(h), with the line of fit in solid black. The mean and standard deviation of the fitting parameters were calculated as the ROIs were moved along the red dashed lines. Although several intensity distributions from the regions with gingivitis were more skewed toward the lower intensity compared to the healthy conditions, the spatially averaged ratio (α/β) from a total of eight different sites with gingivitis were not significantly different from a total of three healthy sites. In addition, note that both the original intensity distribution and the normalized intensity distribution by the maximum intensity within the ROI yielded the similar statistical results. This could be due to the spatial averaging of the fitting parameters and the limited number of subjects. Nonetheless, this texture analysis allows for the assessment of the effective tissue scatterer density and to quantify the homogeneity of the tissue texture using the standard deviation of the alpha/beta ratio in neighboring ROIs.

### 3.4 Longitudinal Tracking of Dental Plaque Distribution

**Fig. 5:**
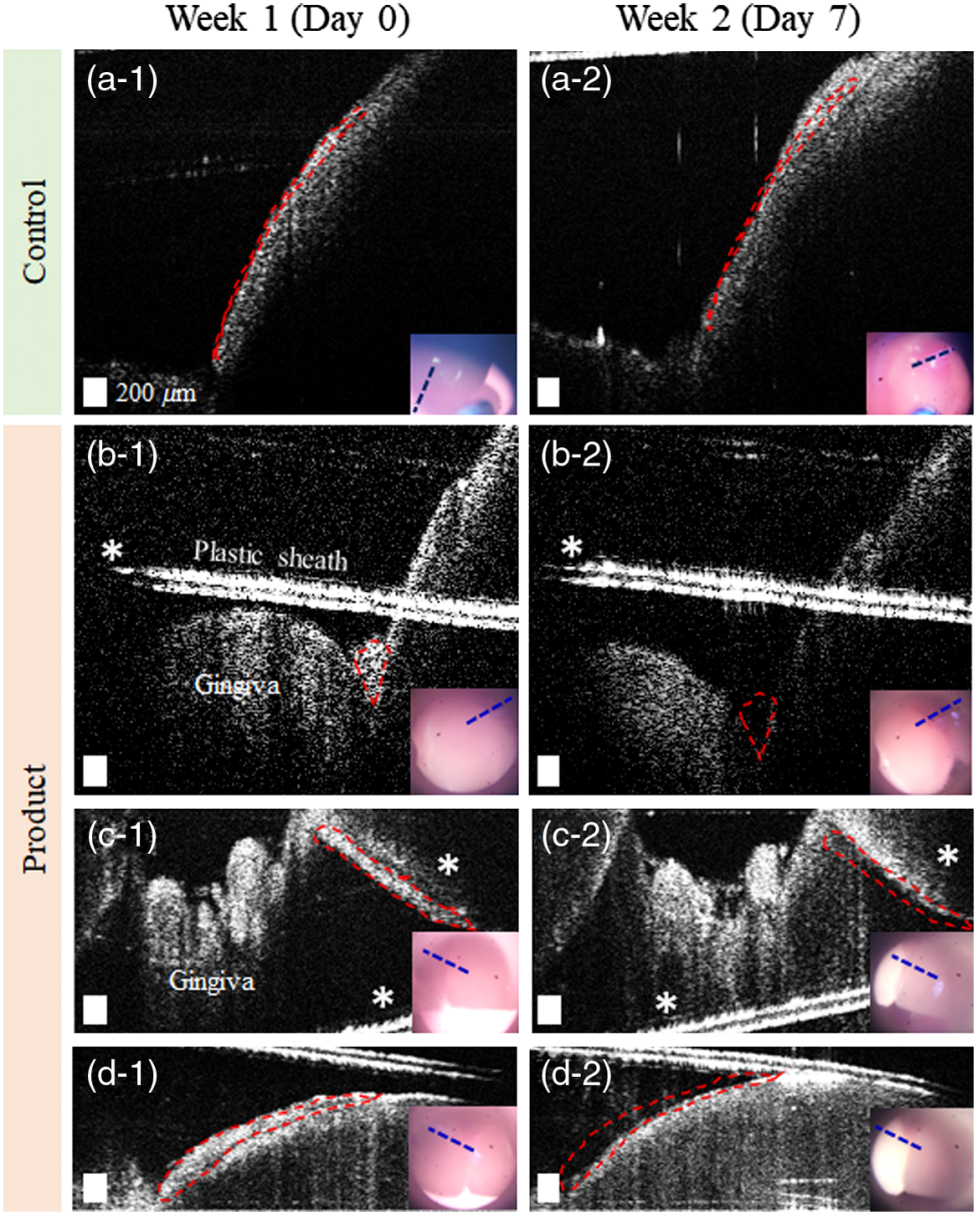
Longitudinal monitoring of dental plaque with OCT. Representative images comparing the plaque distribution after 1-week use of oral hygiene products for (a) the control group and (b)–(d) the product group using the oral hygiene products. The red dashed lines indicate an approximate region of the plaque on week 1 (baseline), which is replicated in the week 2 image to highlight interval changes. The inset images represent the corresponding surface video images, with the blue dashed lines indicating the scanned regions. (a) was obtained from the buccal side of a premolar, (b) was from a canine, and (c) and (d) were acquired from incisors. Scale bars represent 200  μm. Asterisks indicate a region with mirror artifacts.

In order to investigate the feasibility of OCT as a longitudinal monitoring tool, OCT images were acquired again after 1-week use of oral hygiene products (toothpaste, power toothbrush, mouth rinse, and floss), or no use of products (control). The anatomical features, shapes, and curvatures visualized in the surface images (CCD) and the OCT images were used to determine image co-registration. In Fig. 5(a), a representative subject in the control group (no brushing performed, n=5) showed an increased amount of dental plaque after 1 week. In comparison, for a subject in the product group (n=5) instructed to perform regular brushing, the thickness of the dental plaque decreased after 1 week (shown in Figs. 5(b)–5(d). These representative results show that OCT images can be potentially utilized to monitor the distribution of dental plaque when images are co-registered longitudinally over time. Conceivably, the absolute comparison in the amount can be made with the systematic acquisition of volumetric imaging in the future. There were no notable structural changes associated in the gingival conditions, which may be due to the relatively short time interval between imaging, and the limited spatial information.

## 4 Discussion and Conclusion

In this study, a handheld portable OCT system was developed to perform longitudinal dental plaque and gingiva imaging in a clinical setting. There have been several studies that have investigated OCT for the assessment of gingiva and periodontal structures *in vivo*.^23^^,^^24^^,^^36^^,^^39^ For example, Fernandes et al.^23^ used a commercial OCT system to measure the gingival sulcus *in vivo* and compared image data with the traditional methods for healthy individuals. More recently, Le et al.^24^^,^^40^ utilized swept-source OCT and OCT angiography to assess human gingiva and showed different vascular arrangements and features in gingival inflammation. Although these studies show the potential of OCT for detecting microstructures in gingiva, most studies were performed in laboratory settings with bulky imaging systems and mounted optics or probes, limiting the accessibility to posterior teeth and the lingual sides of teeth.

This study explored the versatility and the clinical feasibility of a handheld OCT system to assess dental plaque and gingiva. The handheld OCT probe was operated by trained dental hygienists without prior knowledge of OCT, emphasizing the ease-of-use of the system. The results demonstrate that OCT can visualize the presence of dental plaque at various locations, such as on the surface of the enamel, at the junction between the enamel and gingiva, and between neighboring teeth. In addition, interchangeable probe attachments enabled imaging not only of the frontal side of anterior teeth, but also of posterior teeth and the lingual sides of teeth, where accumulation of plaque is most common.^41^ As many periodontal diseases originate from dental plaque, accurately locating plaque is crucial not only for effective removal, but also for preventing further development of chronic periodontal abnormalities and conditions. With the potential of OCT for longitudinally monitoring plaque, OCT may be helpful for examining changes in the distribution of plaque in therapeutic studies.

Although the averaged fitting parameters from the texture analysis did not show a statistical significance in this preliminary study, the selected sites with severe gingivitis showed a lower intensity distribution and a lower ratio (α/β), indicating the smaller number of scatterers in tissue (Fig. 4). OCT may be more beneficial as a detailed depth-resolved evaluation tool for gingiva after a suspicious region is visually determined from the surface appearance. In addition, the depth-resolved information, such as ulcerative lesions and α/β from the texture analysis, may be possible to reveal early indications of inflammation below the gum line, which may not be visually apparent. The small number of subjects is a limitation of the study. The possibility of type II errors in hypothesis testing implies that there may exist statistical differences in the α/β ratio with a greater sample size. Based on the mean and standard deviation of the α/β ratios from this study, the sample number of >15  in each group would be necessary for more accurate comparison with a statistical power of 0.80 and p=0.05.

A detailed discussion on several challenges from this study can provide valuable perspective for future studies in dental OCT applications. First, while operating the handheld probe in contact mode enhanced the stabilization for imaging, the natural curvature of the teeth resulted in slippage of the imaging probe. With intrinsic limitations of OCT in terms of imaging depth and FOV, the imaging capability and quality, such as SNR, largely depended on individuals’ teeth alignment, especially the canine teeth. Second, a sterile plastic sheath with low light reflectivity was used for efficient and rapid sanitization of the probe between subjects. However, the plastic sheath tightly wrapped around the probe caused more difficulties in effectively using the probe for non-expert OCT users and degraded image quality in some cases. For example, as the teeth were in contact with the probe, the tightened sheath became loose, creating non-flat images of the sheath in OCT. In addition, adjusting the lens tube became inconvenient when the probe was covered with a plastic sheath. In the future, reusable probe attachments that can be autoclaved and sterilized between subjects can be utilized as an alternative, as image quality can both be preserved and be more consistent. Finally, although the buccal and lingual sides of the first molars were captured with the probe attachment and the 45-deg mount, an increase in the length and a decrease in the diameter of the probe attachment, or an endoscope-based system, can further enhance the flexibility for capturing different regions from the posterior teeth, especially for the occlusal sides and the molars.

In this study, OCT and CCD images were used to confirm that the images were taken around the same location. However, questions on image co-registration (both in the transverse plane and in depth) remained, which could be one of the reasons for less significant statistical results. Although OCT could reveal unique tooth curvature in depth, the FOV of the CCD camera was limiting, covering slightly more than one adult tooth, with limited features. This imposed challenges for precisely defining regions of interest, especially for the users (dental hygienists) who were not familiar with interpreting OCT images. Therefore, greater camera FOV for the simultaneous visualization of multiple teeth would be vital to guide the OCT beam, as well as to ensure image registration for longitudinal studies. Using a tooth cover or a bite block and marking with ink that is visible in the near-infrared wavelengths of OCT may be effective to accurately register the locations, as most marker inks are invisible in OCT.^42^ Although the OCT system can acquire 2D images in real time, a trained non-OCT expert spent the most imaging time on locating the imaging site and adjusting the probe angle to ensure good image quality. This demonstrates another limitation of the practical application of OCT in dentistry, as also supported by a previous study.^23^

In the future, many limitations can be overcome by acquiring high-speed volumetric images, developing a larger FOV with bright illumination for better surface visualization, and avoiding the use of a plastic sheath. In addition, image co-registration in depth can also be enhanced by using swept-source OCT or full-range OCT, which extends the imaging range. The wavelengths around 800 nm were utilized because this handheld OCT system was initially designed as an imaging tool for general primary care applications, such as for skin, ear, eye, and oral cavity imaging.^43^ If the targeted application includes both soft and hard tissues in the oral cavity, the use of longer wavelengths can improve the imaging depth, but with a compromised resolution. Furthermore, the use of polarization sensitive OCT may help differentiate enamel from dental plaque, as the hydroxyapatite crystals in enamel are known to be birefringent.^35^ This study demonstrated the versatility of a handheld OCT imaging system and its feasibility to provide depth-resolved information in gingiva and dental plaque longitudinally in a clinical setting. Ongoing improvements in methods and probe designs will enable more practical clinical instruments for evaluating efficacy of dental treatments and assessing dental health.

## Data Availability

The data and the associated code that support the findings of this study are available from the corresponding author upon reasonable request and through a collaborative research agreement.
